# Radiographic Inspection of Carbon Fiber-Reinforced Polymer Composites (Laminates) with Epoxy and PEEK Binders After Impact and Subsequent Compression Loading

**DOI:** 10.3390/polym16233262

**Published:** 2024-11-23

**Authors:** Pavel V. Kosmachev, Dmitry Yu. Stepanov, Anton V. Tyazhev, Alexander E. Vinnik, Alexander V. Eremin, Oleg P. Tolbanov, Sergey V. Panin

**Affiliations:** 1 Microelectronics of Multispectral Quantum Introscopy Laboratory of the R&D Center “Advanced Electronic Technologies”, National Research Tomsk State University, 634050 Tomsk, Russia; sdu@ispms.ru (D.Y.S.); antontyazhev@mail.ru (A.V.T.); evg2v@mail.ru (A.E.V.); top@mail.tsu.ru (O.P.T.); 2 Laboratory of Mechanics of Polymer Composite Materials, Institute of Strength Physics and Materials Science of Siberian Branch of Russian Academy of Sciences, 634055 Tomsk, Russia; ave@ispms.ru (A.V.E.); svp@ispms.ru (S.V.P.)

**Keywords:** fiber-reinforced polymer composite, laminate, radiographic inspection, impact damage, image processing, flaw detection, image quality, signal-to-noise ratio

## Abstract

An approach to detecting discontinuities in carbon fiber-reinforced polymers, caused by impact loading followed by compression testing, was developed. An X-ray sensor-based installation was used, while some algorithms were developed to improve the quality of the obtained low-contrast radiographic images with negligible signal-to-noise ratios. For epoxy/AF (#1) composite subjected to a “high-velocity” steel-ball impact with subsequent compression loading, it was not possible to detect discontinuities since the orientation of the extended zone of interlayer delamination was perpendicular to the irradiation axis. After drop-weight impacts with subsequent compression loading of epoxy/CF (#2) and PEEK/CF (#3) composites, the main cracks were formed in their central parts. This area was reliably detected through the improved radiographic images being more contrasted compared to that for composite #3, for which the damaged area was similar in shape but smaller. The phase variation and congruency methods were employed to highlight low-contrast objects in the radiographic images. The phase variation procedure showed higher efficiency in detecting small objects, while phase congruency is preferable for highlighting large objects. To assess the degree of image improvement, several metrics were implemented. In the analysis of the model images, the most indicative was the PSNR parameter (with a S-N ratio greater than the unit), confirming an increase in image contrast and a decrease in noise level. The NIQE and PIQE parameters enabled the correct assessment of image quality even with the S-N ratio being less than a unit.

## 1. Introduction

“The hardest thing of all is to find a black cat in a dark room, especially if there is no cat”. Confucius.

The development and implementation of advanced inspection techniques to detect discontinuities/damage in polymer composites (both particulate and reinforced with long or continuous fibers) and monitoring their structural integrity are relevant tasks nowadays, including for those with matrices/binders based on high-performance polymers (HPP) [1,2]. Since fiber-reinforced composites are multi-level organized by their definition [3,4,5], different methods should be applied to detect damage/flaws of various scales [6,7].

For the industrial non-destructive testing (NTD) of carbon fiber-reinforced polymers (CFRPs), many techniques are deployed, differing in sensitivity, spatial resolution, type, orientation, and the dimension of detected discontinuities, permissible sizes of objects under inspection, as well as accessibility of inspection. As a result, they enable the detection of certain types of discontinuities, depending on both features of the CFRPs and production routes for their fabrication [8].

The high structural heterogeneity of CFRPs should be emphasized, which, during non-destructive testing (NDT) using active methods, causes noticeable scattering, reflection, and ultimately the attenuation of the probing signal. On the other hand, the dimensions of flaws and discontinuities, which critically affect both the strength and structural integrity of CFRPs (with low crack resistance by nature), can be quite small. Finally, one of the most pressing challenges is barely visible impact damage (BVID), in the detection of which conventional NDT methods are ineffective in many cases [9,10].

Nevertheless, many aspects of the application of various NDT methods for detecting flaws in both laboratory samples and structural elements are discussed nowadays. For U-sonic inspection, as an example, some approaches based on the use of phased antenna arrays [11] and nonlinear methods of action, which allow for more sensitive detection of various types of damage [12], are widespread. Acoustic NDT methods are primarily classified over probing frequency. The guided waves method allows for the monitoring of the formation of discontinuities in structural elements with dimensions of a single meter due to a lower attenuation of elastic vibrations (with a characteristic frequency of hundreds of kHz) than that in U-sonic inspection [13]. Vibration analysis is another low-frequency method, enabling the detection of delamination in plates, beams, and laminated composites [14,15,16]. For NDT of extended parts and structures, acoustic emission is applied since the fracture of reinforcing fibers gives rise to signals of sufficiently great amplitudes [17]; however, it is hardly applicable to the detection of individual defects.

Interferometric/shearography methods, which are highly sensitive to the development of out-of-plane deformations, can be successfully applied to detect impact discontinuities of various sizes [18,19]. Thermal (infrared) inspection methods are also used if the thermophysical properties of materials and their inclusions/voids differ significantly [20,21]. Recently, some research results have been reported on testing CFRPs through terahertz inspection, which is suitable for detecting impact damage in thick products [22,23].

For the reason of high spatial resolution, as well as penetration ability, X-ray radiography is one of the most effective approaches to monitoring structural integrity and detecting discontinuities [24], including inverse Compton scattering [25]. In addition, scanning with angular cone beams [26] enables the obtainment of data close to tomographic results [27]. The use of X-ray computed tomography (CT) [28,29,30] and μCT [31,32,33,34] opens up broad prospects in terms of identifying and quantitatively characterizing the internal structure, manufacturing defects, and operational damage in CFRPs. However, these methods are quite difficult to use for NTD of extended structures, particularly with limited access.

Over the past 25 years, the structural health monitoring (SHM) approach has been actively developing. It simultaneously implements several NDT methods, including the above-mentioned ones [35,36,37]. The combined use of different NDTs fused with the implementation of the digital twin concept makes SHM possible to provide a more comprehensive strategy for assessing structural integrity, especially in intricate composite materials. However, this approach does not detect individual discontinuities, but it indicates the onset of alarm or fault conditions, allowing for timely decisions regarding changes in operating modes or repairing deteriorated structures. Within the SHM framework, the fiber Bragg grating effect is implemented [38], lightening optical fibers, increasing their sensitivity, and eliminating the need for power supply. One of the varieties of this approach is optical backscatter reflectometry [39].

Considering the above, it becomes obvious that the use of X-ray NDT methods remains one of the most effective and reliable approaches to monitoring the structural integrity of CFRPs. However, existing X-ray diagnostic equipment has largely exhausted its capabilities due to both the low X-ray contrast of investigated objects and the small sizes of discontinuities capable of critically affecting the safe operation of such products [40]. The development of a new generation of digital facilities with high spatial and energy resolution will allow for solving a wider range of industrial tasks.

The interest in developing X-ray sensors for NDT of CFRP is caused by the significant dependence of the strength properties on the characteristics of interfaces between both phases and layers. Given that reinforced polymer composites are a continuous set of interfaces, their fast full-thickness inspection is necessary to ensure the reliable performance of components and structure elements.

The detection of flaws in products made from such composites under various operating conditions is challenging as well (for example, after hygrothermal/freeze-thaw exposure [41] or cyclic and/or impact loading [42]). In such cases, the challenge is caused by the small opening of BVID (from tens of micrometers to fractions of millimeters), as well as the large cross-sectional sizes (up to units and even tens of millimeters) and lengths (fractions and units of meters) [43]. For this reason, the use of digital systems based on semiconductor X-ray sensors opens up wide prospects for non-destructive testing. Therefore, the task of developing X-ray diagnostic methods for monitoring the structural integrity of CFRPs should be solved comprehensively. For both hardware and software solutions, it is necessary to apply laboratory conditions, which ensure registration high-quality radiographic images (with low noise levels) and their processing (enhancement). At the same time, CFRP samples with known discontinuities (including BVID) should be used for investigations [44].

According to the generally accepted codes, NDT devices are calibrated using reference samples with discontinuities obviously larger than the spatial resolution of the sensors (as an example, U-sonic inspection, where the dimensions of typical detectable flows are several millimeters). In contrast, the use of commercially available digital semiconductor X-ray detectors does not imply spatial resolutions of less than 50 μm. In addition to optimizing the radiographic inspection parameters (energy, exposure, focal length, and quantum accumulation mode), which, however, is beyond the scope of this study, the development of effective algorithms for processing the obtained images is crucial. The reason is low radiation doses and low signal-to-noise (S-N) ratios, which do not enable the recording of contrast images for CFRP. Improving the quality of X-ray images by increasing spatial and energy resolutions at the hardware level often requires large expenditures of time and resources. This issue can be solved through digital processing of low-contrast X-ray images, obtained with conventional detectors.

This study aimed to develop an approach to detecting discontinuities in CFRPs, induced via impacts followed by compression testing, with the use of an X-ray sensor-based installation and the processing of radiographic images. The paper is structured as follows: Section 2 briefly describes the installation used to acquire radiographic images. Section 3 explains approaches to processing low-contrast images with a low S-N ratio. Section 4 describes the samples used for testing and the results obtained. Preceding the conclusions, Section 5 contains a brief discussion, as well as suggested shortcomings and future prospects.

## 2. Installation for Radiographic Inspection of Low-Contrast Objects

The key up-to-date radiographic installations for scientific, industrial, and medical applications are based on a two-stage conversion of X-ray quanta (after passing through the object under study/inspection). In these cases, a scintillator converts the radiation quanta into ultraviolet or visible spectra, while the secondary quanta are transformed into electric current pulses with photodetector matrices, charge-coupled devices (CCDs), or thin-film transistors [45,46,47]. This double conversion reduces the detective quantum efficiency (DQE), as well as the S-N ratio and the quality of X-ray images.

Over the last decade, the worldwide scientific community has been actively developing semiconductor multi-element detectors [48,49,50]. This process was triggered by the need to carry out physics experiments at the Large Hadron Collider in the 1990s. Microstrip and pixel detectors based on ultra-pure single-crystal silicon to record the coordinates of high-energy charged particles have been developed and manufactured by several Japanese and American companies. However, silicon is absolutely transparent to synchrotron, X-ray, and gamma radiation in the quantum energy range above 15 keV. This has spurred the development of fundamental scientific and technological fields aiming to improve coordinate detectors based on complex semiconductors with a high atomic number, *Z*. For the X-ray energy range above 10 keV, the most promising semiconductor materials are GaAs and Cd(Zn)Te [51,52,53,54,55]. In terms of both technological characteristics and the price–performance ratio, GaAs is superior to Cd(Zn)Te since its cost is significantly lower (by several times), it is more technologically advanced, it has higher property homogeneity, and it achieves a low defect level [56,57,58,59].

Despite the obvious promise of GaAs detectors, a commercial Si-based one was used in this study for the registration of radiographic images. This choice was driven by its greater S-N ratio compared to a similar GaAs sensor. The lack of an available GaAs detector with comparable pixel sizes (above 200 µm) was another reason, in addition to the necessity of ensuring the comparability of the obtained data with the results of related studies, primarily in terms of both spatial resolution and the S-N ratio, as well as the task of developing the software embedded into the installation.

**Figure 1:**
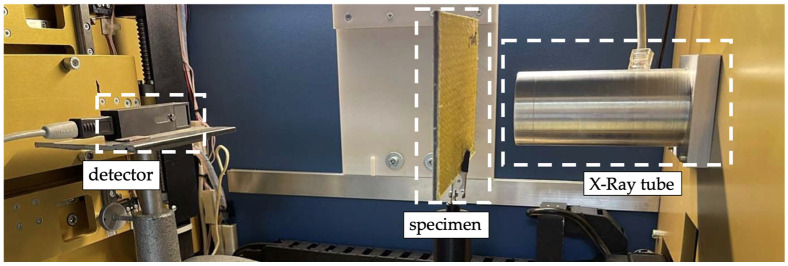
A scheme of the inspection process using the laboratory radiographic setup.

Measurements were carried out using a laboratory setup (Figure 1), the key components of which were as follows:

- An “XRB011” X-ray source (Spellman High Voltage Electronics Corporation, New York, NY, USA) based on a “1000 Glass” microfocus tube (Oxford Instruments, Abingdon, UK) with a tungsten anode and an X-ray matrix detector was operated in the quantum counting mode. The technical specifications of the X-ray source are as follows: a tunable range for the X-ray tube current of 1 ÷ 700 μA with an adjustment step of 10 μA and a range of accelerating voltage 35 ÷ 80 kV with a step of 5 kV. The average value of the X-ray focal spot size was 50 μm, and the radiation divergence angle was 40 degrees. The X-ray beam was collimated with a lead collimator to implement a 25 mm-diameter spot size.
- A silicon sensor of 1 mm in thickness was used in the detector. The sensor topology was a 256 × 256-pixel matrix with a pixel pitch of 55 μm.
- Radiographic inspections were carried out under an accelerating voltage of 35 kV and a current of 500 μA. The exposure time varied in the range of 60–180 s.

Original radiographic images were subjected to the “Flat-field correction” operation [60] in order to compensate for the technological spread of the sensitivity of each sensor pixel, the gain in the corresponding electronics channel, and the noise of the measuring path. For each image, the exposure time was chosen so that the total number of recorded quanta was about 10,000 per image, corresponding to a relative quantum flow noise value of 1%.

## 3. Processing of Radiographic Images

The key issues solved in improving the quality of the radiographic images included noise suppression, contrast enhancement, and/or target detection for (low-contrast) object contours on them. The features of the images were the presence of high-level irregular and regular noise caused by spatial recording unevenness. While the second challenge was solved using the “Flat-field correction” procedure, the first challenge was typically overcome by increasing the radiation power and/or the registration time. However, such a solution was not always possible for some practical reasons, so a number of highly efficient digital methods of image processing were implemented.

### 3.1. Pre-Processing

The pre-processing of radiographic images comprises a wide range of various routines: denoising, cropping, thresholding, binarization, morphology transformation, histogram equalization, etc. [61,62,63]. Their applicability is case-sensitive and depends on the noise pattern, as well as the efficiency of every particular procedure. Upon a preliminary analysis of the registered radiographic images, four successive conversions were selected and carried out:

1. The “Flat-field correction” procedure. It is a digital imaging technique for mitigating the pixel sensitivity of an image detector and distortions in the X-ray path. It referred to the process of compensating for the different gains and background currents in the detector. Once the detector was properly adjusted for the flat field, uniform output signals were registered without any systematic error due to beam inhomogeneities, variations in the detector response gain, charge-transfer losses, charge trapping, or variations in the readout performance [64,65].
2. Dead pixel reassignment (thresholding). During the data recording process, some image pixels may be lost (zeroed). To mitigate their negative impact on subsequent processing, they are replaced by the median value of their proximity.
3. Adaptive median or bilateral filtering. This technique is applied to reduce noise levels and, in particular, eliminate random outliers [66,67,68,69]. The efficiency of various filtration algorithms as a function of noise distribution is reported elsewhere in [70,71]; however, it is beyond of the scope of this paper.
4. Radiographic marker reassignment. Special radiographic markers, typically with a significantly higher absorption coefficient, are placed on radiographic images for more accurate localization of the imaging area. These markers interfere with subsequent image processing and the analysis of the objects under study, and their influence needs to be reduced. Since the intensity of their image falls within a lower value range, it is proposed to replace all pixels within this identified range with the average value from the intensity range of the object being examined.

### 3.2. Improving the Quality of Radiographic Images

Object contours’ extraction from images can be achieved using various methods. Early methods were based on the evaluation of image gradients. They included algorithms for the gradient binarization of images (for example, the Roberts, Prewitt, Sobel, and Canny operators [72]). A family of algorithms based on stochastic principles and optimization methods, known as the ant colony system (ACS), has also been developed [73,74]. All of these methods are quite effective for processing photographs with a high signal-to-noise ratio, but their application is ineffective for processing radiographic images due to the presence of intense interference.

Methods with high noise immunity should include not only the direct analysis of the image pixels’ variability but also noise suppression procedures. A number of algorithms were developed, which are based on the following property of the 1D Hilbert transform: a step-function is transformed into a unimodal function. The problem of its extending to the transformation of 2D grayscale images is widely discussed by many researchers [75,76,77,78,79]. In this study, the radiographic images were processed using the phase variation method and congruency method.

#### 3.2.1. The Phase Variation Method

**Figure 2:**
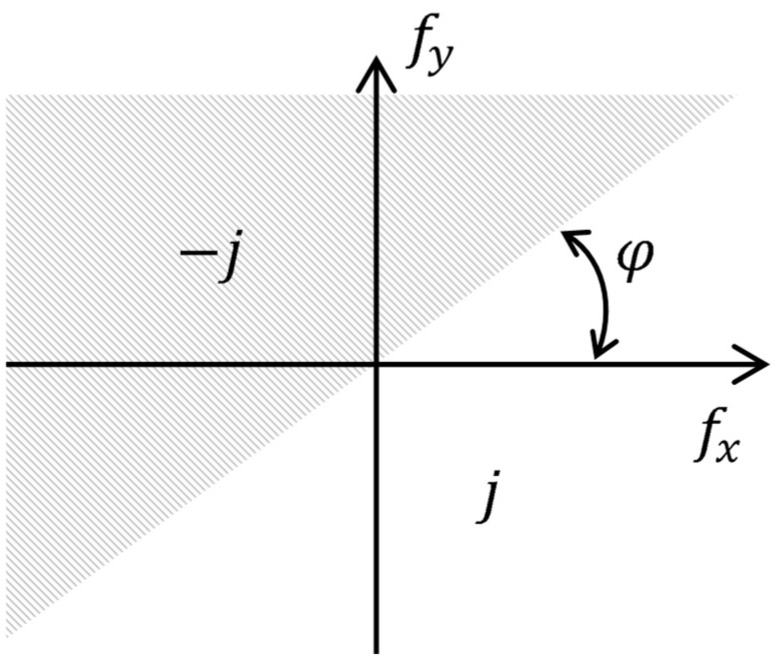
The frequency response of the 2D directional Hilbert filter.

If a grayscale image was represented by a 2D function *I*(*x*, *y*) of spatial variables *x* and *y*, then the following transformation could be taken as an analogue of the 2D Hilbert transform:

(1) $$I(f_{x},f_{y};\varphi )=\iint_{-\infty }^{\infty }I\left(x,y\right)e^{-j\frac{\pi }{2}sign\left({\sin\left(\varphi \right)f}_{x}+\cos\left(\varphi \right)f_{y}\right)}e^{-j2\pi \left(xf_{x}+yf_{y}\right)}dxdy,$$

where $f_{x}$ and $f_{y}$ were corresponding spatial frequencies, and φ was a slope angle of the transformation. This expression could be interpreted as directional filtering in the 2D frequency domain with the following characteristic (Figure 2):

(2) $$H_{G}\left(f_{x},f_{y};\varphi \right)=e^{-j\frac{\pi }{2}sign({\sin\left(\varphi \right)f}_{x}+cos(\varphi )f_{y})},$$

or in polar coordinates:

(3) $$H_{G}\left(\omega ,\theta ;\varphi \right)=\left\{\begin{array}{c} j,\theta <\varphi ,\;\\ 0,\theta =\varphi ,\\ -j,\theta >\varphi , \end{array}\right.$$

where $\omega =\sqrt{f_{x}^{2}+f_{y}^{2}}$, $\theta =tg\left(\frac{f_{y}}{f_{x}}\right)$ were the frequency polar coordinates. The application of this filter to image processing enabled the highlighting of the object contours with line slope angles close to the φ value.

To obtain an image of the object contour with an arbitrary direction, multiple filtering with different $\varphi_{k}=k\frac{\pi }{N}$ angles and a summing up the modules of their output was implemented:

(4) $$V\left(x,y\right)=\sum_{k=0}^{N-1}\left|F^{-1}\left\{I(f_{x},f_{y};\varphi_{k})\right\}\right|,$$

where $F^{-1}\left\{\ldots \right\}$ was the inverse 2D Fourier transformation. This expression was called the phase variation, in an analogy for the phase congruence.

Under intense interference conditions, the filter could be ineffective. So, the obvious solution to the noise immunity problem was to combine it with known filters to suppress random interference (for example, with low-pass, Butterworth, Gaussian, elliptical, or bandpass ones). In this study, the 2D symmetric Gaussian filter was used:

(5) $$H_{\sigma }\left(\omega ,\theta ;\sigma \right)=e^{-\frac{\omega^{2}}{2\sigma^{2}}},$$

where σ determined the bandwidth of the filter according to the ω polar frequency.

**Figure 3:**
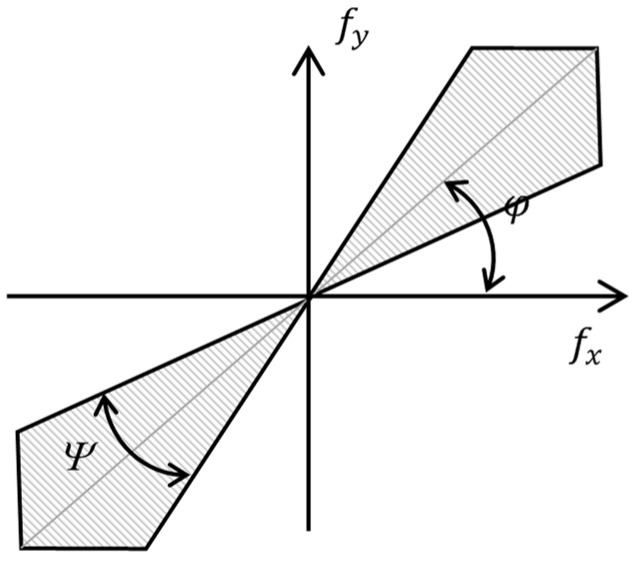
The frequency response of the fan filter.

Another technique that improved the efficiency of detecting discontinuous boundary contours was to limit the filter bandwidth to the *θ* angle. One of the simplest ways to apply this limitation was to combine 2D filters with a fan filter (Figure 3):

(6) $$H_{\Psi }\left(\omega ,\theta ;\varphi ,\Psi \right)=\left\{\begin{array}{l} 1,\varphi -\Psi /2<\theta <\varphi +\Psi /2,\\ 0,\;otherwise, \end{array}\right.,$$

where φ and *Ψ* determined the direction and the solution of the fan filter, respectively.

Considering the above, the improved phase variation function took the following form:

(7) $$VP\left(x,y\right)=\sum_{k=0}^{N-1}\left|F^{-1}\left\{I(f_{x},f_{y})H_{G}\left(f_{x},f_{y};\varphi_{k}\right)H_{\sigma }\left(f_{x},f_{y};\sigma \right)H_{\Psi }\left(f_{x},f_{y};\varphi_{k}+\frac{\pi }{2},\right)\right\}\right|.$$

In such calculations, a restriction was imposed on the fan filter solution:

(8) $$\frac{\pi }{N}\leq \Psi \leq \frac{2\pi }{N}.$$

The phase variation algorithm includes the following steps:

1. The 2D Fourier transform of a radiographic image $I\left(f_{x},f_{y}\right)=F\left\{I(x,y)\right\}$;
2. Calculation of the Gaussian filter phase response (5) succeeded by multiplication with a 2D Fourier pattern of the image;
3. The calculation of the Hilbert filter (3), as well as the fan filter (6) phase responses for the specified angle, $\varphi_{k}$;
4. The multiplication of phase responses of (3) and (6) with the calculation result obtained in step 2;
5. The calculation of the modulus of the inverse 2D Fourier transform from the result obtained in step 4;
6. Repetition of steps 3–5 for k=0,..,N;
7. The calculation of the sum of results from step 5.

#### 3.2.2. The Phase Congruency Method

**Figure 4:**
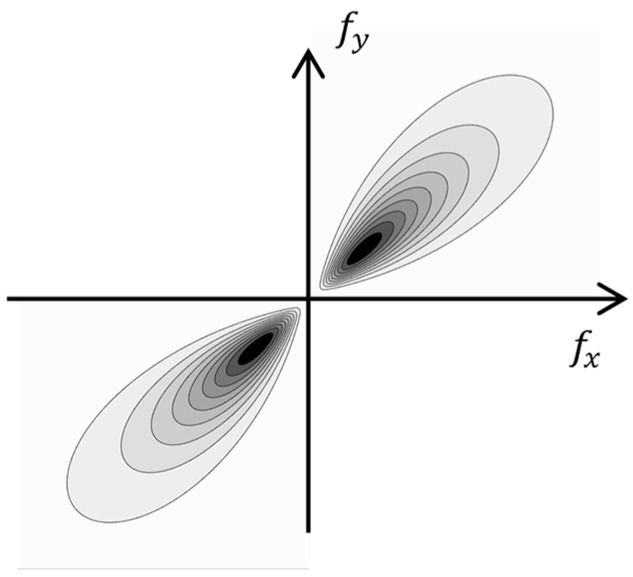
The 2D logarithmic Gabor function.

The studies [80,81,82] proposed using a spiral phase filter or wavelet analysis with the 2D logarithmic Gabor functions (Figure 4):

(9) $$G_{s,k}\left(\omega ,\theta \right)=e^{-\frac{{ln}^{2}\left(\frac{\omega }{\omega_{k}}\right)}{2{ln}^{2}\left(\sigma_{\omega }\right)}}e^{-\frac{{\left(\theta -\theta_{k}\right)}^{2}}{2\sigma_{\theta }^{2}}},$$

where ω and θ were the polar coordinates in the frequency domain, $\omega_{k}$ and $\theta_{k}$ determined the order and the direction of the transformation, and $\sigma_{\omega }$ and $\sigma_{\theta }$ could be interpreted as effective filter passbands.

Phase congruency was calculated by enumerating the order and direction of the Gabor functions and summing the filtering results. For example, the expression of the phase congruency with an *ε* noise compensation and a threshold for small *T* values was as follows:

(10) $$PC\left(x,y\right)=\frac{\left\lfloor \sum_{s=0}^{M-1}\sum_{k=0}^{N-1}\left|F^{-1}\left\{I(f_{x},f_{y})G_{s,k}\left(f_{x},f_{y}\right)\right\}\right|-T\right\rfloor }{\left|\sum_{s=0}^{M-1}\sum_{k=0}^{N-1}F^{-1}\left\{I(f_{x},f_{y})G_{s,k}\left(f_{x},f_{y}\right)\right\}\right|+\varepsilon },$$

where $\left\lfloor z\right\rfloor$ represented the positive part function of *z*. The algorithms, as well as a computer code for phase congruency, are reported elsewhere in [80].

### 3.3. Image-Quality Estimates

The following metrics are among the most widespread and frequently used [83,84,85]:

- Peak signal-to-noise ratio:

(11) $$PSNR=10log\left[\frac{I_{max}^{2}}{MSE}\right]$$

where $I_{max}$ denotes the maximum value of image brightness, and *MSE* indicates a mean square error of brightness. The better the quality, the higher the *PSRN* (measured in dB);

- A structural similarity index:

(12) $$SSIM=\left(\frac{R_{IJ}}{\sigma_{I}\sigma_{J}}\right)\left(\frac{2m_{I}m_{J}}{m_{I}^{2}+m_{J}^{2}}\right)\left(\frac{2\sigma_{I}\sigma_{J}}{\sigma_{I}^{2}+\sigma_{J}^{2}}\right)$$

where m, σ, and R represent the mean brightness values, variance, and a correlation coefficient, correspondently. Its value is limited within the range of [0, 1], where zero means no similarity, while a unit corresponds to the complete identity.

Estimates ((11)–(12)) require a pristine (etalon) image, *J*. However, there is no pristine image in the processing of real images, first of all due to the presence of noise.

Recently, some novel “absolute” metrics have been developed. They do not require a pristine image, while they are based on expert estimates. The following ones were used in the study:

- The natural image quality evaluator (NIQE) measures the quality of images with arbitrary distortion. In doing so, a distance between the NSS (natural scene statistics/NSS)-based features is calculated from a current image to the features obtained from an image database used to train the model. The features are modeled as multidimensional Gaussian distributions. Thus, the NIQE is opinion-unaware and does not use subjective quality scores [86].
- The perception-based image quality evaluator (PIQE). The PIQE algorithm is opinion-unaware and unsupervised; thus, it does not require a trained model. PIQE measures the quality of images with arbitrary distortion being similar to the NIQE. Thus, the PIQE estimates block-wise distortion and measures the local variance in perceptibly distorted blocks to estimate the quality score [87]. The values of PIQE in the range of 0 ÷ 20 correspond to excellent image quality, while its values of 81 ÷ 100 are qualified as bad image quality.

## 4. Results

### 4.1. The Methodology for Recording the Experimental Data

To test the laboratory hardware and software installation, three types of layered composites (laminates) were used. The samples of the three composites had a rectangular plate shape with dimensions of 100 × 150 mm and, for impact loading, were clamped on a fixture base with a window of 75 × 125 mm.

Composite #1 was subjected to a point-impact action (with a striker in the form of a steel ball) flying from a gas gun at a speed of ~90 m/s and an energy of ~49 J. Composite #2 and composite #3 were tested using an Instron Ceast 9340 drop-weight testing machine (Instron, Norwood, MA, USA) at a speed of ~3.5 m/s and an energy of 20–43 J. Details of the sample fabrication and the results of the residual compressive strength evaluation after the impacts are described below.

#### 4.1.1. Composite #1

The sample was made from two types of prepregs (with aramid fabric or carbon unidirectional tape) and an epoxy binder. In this sample, a quasi-isotropic layup was implemented with a 50/50 component ratio, namely ((−45F/90F)_2_/(45/0/−45/90)_2_)_S_. The aramid prepreg type was “AA285”, with plain weave, a fabric density of 170 g/m^2^, and a monolayer thickness of 0.27 mm. The carbon prepreg, type “UTS-150-DT190-36F”, consisted of unidirectional “AS4D” fibers without a weft thread. There were two layers of carbon unidirectional tape per single layer of the aramid woven fabric (designated as “F”). The composite is designated as “epoxy/AF” in the text.

The mechanical properties of monolayers obtained in the experiments are as follows: aramid fabric prepregs exhibited a tensile strength of 620 MPa, a compression strength of 167 MPa, and an elastic modulus of 44 GPa, while carbon fiber prepregs demonstrated a tensile strength of 2042 MPa, a compression strength of 1495 MPa, and an elastic modulus of 138 GPa.

The manufacturing process consisted of the manual layup of prepregs in the required order, followed by hot pressing at a pressure of 0.7 MPa and a temperature of 100 °C for 1 h. To complete all polymerization processes and reduce residual stresses in the composite, the plates were subsequently placed in a thermal oven at 100 °C for 24 h. The blank plates, measuring 217 × 152 × 3.2 mm^3^, were then cut using a CNC milling machine equipped with a polycrystalline diamond tool to obtain the final test plates with dimensions of 100 × 150 × 3.2 mm^3^.

Composite #1 (epoxy/AF) was subjected to “high-velocity” (~90 m/s) impact. A steel ball with a diameter of 14.3 mm and a weight of 11.9 g was used as a striker. After impact, its residual compressive strength was measured. The test results are presented in Table 1.

#### 4.1.2. Composite #2

Composite #2 was made with the (45F/90F)_5S_ layup from dry carbon biaxial fabric “CBX-300”, with a weft yarn and epoxy binder “L/GL2”, designated as “epoxy/CF”. It was characterized by a density of 305 g/m^2^. The monolayer of unidirectional fibers exhibits a tensile strength of 1455 MPa, a compressive strength of 1296 MPa, and an elastic modulus of 135 GPa. Testing of the specific properties of the biaxial fabric was not conducted.

The manufacturing process consisted of the manual layup of fabric, followed by the manual deposition of an epoxy binder and rolling to distribute the adhesive, as well as to better impregnate the fibers. Then, the stack was placed in a hot press for 1 h at a pressure of 0.7 MPa and a temperature of 100 °C. To complete all polymerization processes and reduce residual stresses in the composite, the plates were subsequently post-cured in a thermal oven at 100 °C for 24 h. Then, the blanks were cut using a CNC milling machine to the dimensions of 100 × 150 mm.

Unlike the previous case, low-velocity impact testing was carried out with a drop weight, followed by compression testing of the damaged sample [88]. The cylindrical impactor with a hemispherical tip had a weight of 5.4 kg and a diameter of 12.7 mm. The results of both tests are presented in Table 2.

#### 4.1.3. Composite #3

Composite #3 was fabricated with the (90/0/90/0/45/−45/45/−45)_2S_ layup from “Toray Cetex^®^ TC1200 PEEK” (Toray Advanced Composites, Morgan Hill, CA, USA) unidirectional carbon fiber preform and a PEEK binder, designated as “PEEK/CF”. It was tested in the same way as composite #2. The test results are presented in Table 3.

The PEEK/CF prepreg exhibited a tensile strength of 2410 MPa, a compressive strength of 1300 MPa, and an elastic modulus of 135 GPa. The dry prepregs were manually arranged in a stack following the required stacking sequence. The hot press was preheated to 300 °C, after which the stack was placed in the press. The temperature was then increased to 380 °C under a pressure of 0.7 MPa and held for 1 h. Following this, the heating was turned off, and the assembly was cooled to 300 °C. Finally, the blank plate was removed from the press and allowed to cool in air to room temperature.

The performed compression tests were treated as a conventional method for assessing the reduction in the load-bearing capacity of the CFRPs preliminarily subjected to impact loading [89].

In the framework of this study, they enabled an increase in the dimensions of macroscopic damage in each sample to facilitate their subsequent visualization during radiographic inspection since BVID with the spatial resolution of the sensor applied is practically impossible to detect even with this method.

**Figure 5:**
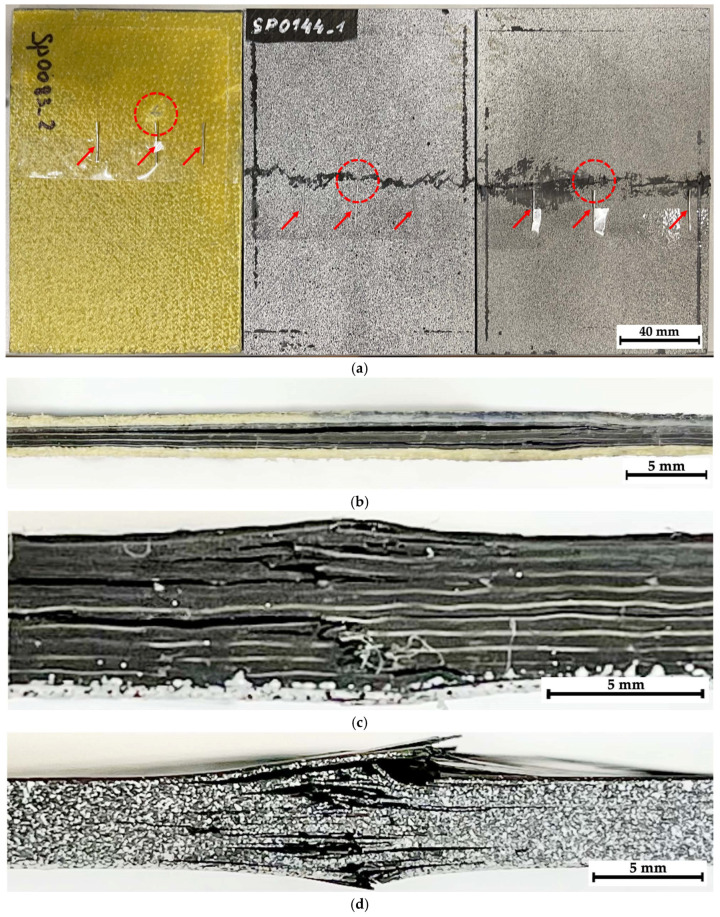
The samples for radiographic inspection: (**a**) A general view in the plane of impact; the dotted line indicates the contact spots with the striker, while the arrows indicate the office staples used as indicator marks. Side views of composites #1 (**b**), #2 (**c**), and #3 (**d**).

General views of the tested samples are shown in Figure 5 (from the impact side, the striker contact spots are indicated). In addition, their side views are presented, illustrating the failure modes after compression testing.

Figure 5a shows that composite #1 (epoxy/AF) experienced significant longitudinal (interlaminar) delamination, corresponding to a low residual compressive strength of 67.7 MPa. In the case of composite #2 (epoxy/CF), the impact loading was characterized by a maximum adsorbed energy of >43 J, but the failure energy was extremely low (~3 J). After compression testing, both longitudinal delamination and transverse cracking were observed (Figure 5c). The residual compressive strength was significantly higher than in composite #1, at 175 MPa. On the contrary, the total energy of ~20 J was low for composite #3 (PEEK/CF), while its failure energy increased up to 10 J. After compression testing, composite #3 was characterized by multiple cracks, which apparently allowed it to absorb a noticeable part of the energy introduced via impact, while the residual compressive strength of ~290 MPa was the greatest among all the studied samples.

**Figure 6:**
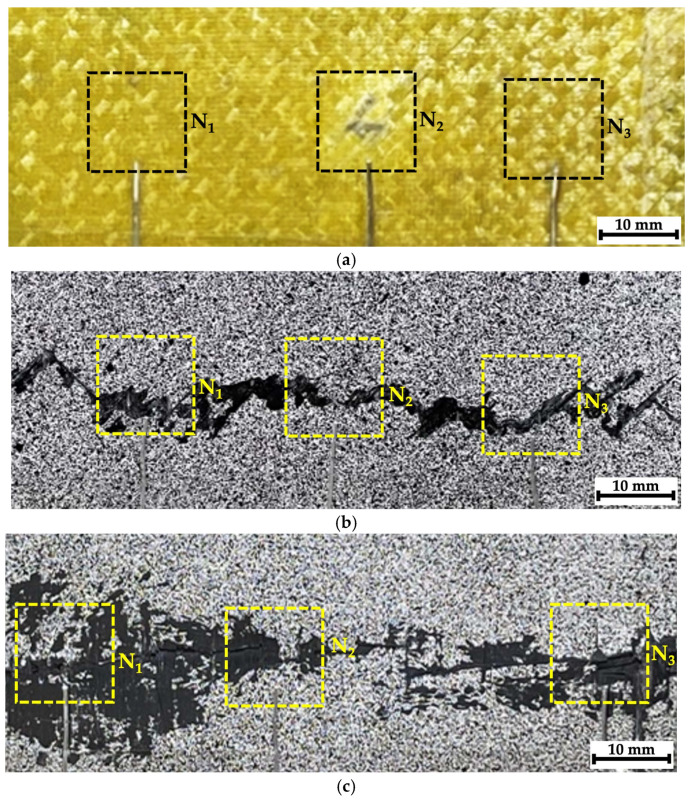
The regions of radiographic inspection of composites #1 (**a**), #2 (**b**), and #3 (**c**).

In Figure 5a, it can be seen that, as a result of the impact on composite #1 (epoxy/CF), a rounded trace was formed on the surface, comparable in size to the diameter of the ball (shown in the red circle). Since the sample was subjected to compression testing after the impact, it was of interest to visualize possible damage in its several areas using the hardware/software installation. For this purpose, three regions were selected, corresponding to the striker contact spots (Figure 6a, region N_2_) and located at approximately equal distances from it (Figure 6a, regions N_1_ and N_3_), for which radiographic inspection was carried out. To facilitate the location goal before the radiographic inspection, office staples were attached to the sample surface with adhesive tape, as shown using the arrow in Figure 5a.

Since the images of the side face of the studied samples shown in Figure 5b–d did not enable an understanding of the distance from the impact spot or from the transverse main crack at which the delamination/fracture processes developed, an additional radiographic inspection was conducted. Within its framework, stitched images (montages/series of adjacent sections) were investigated in all three composites. In these cases, the radiographic images were recorded with overlapping sections to avoid a possible loss of information.

For composites #2 (epoxy/CF) and #3 (PEEK/CF), similar images are shown in Figure 6b,c, with an indication of the regions for the radiographic inspection that were located not only in the center (in fact, the spot of contact with the striker) but also at the sample edges through the main crack (initiated during compression testing). According to the results of the visual analysis, they could correspond to areas that had experienced varying degrees of damage.

**Figure 7:**
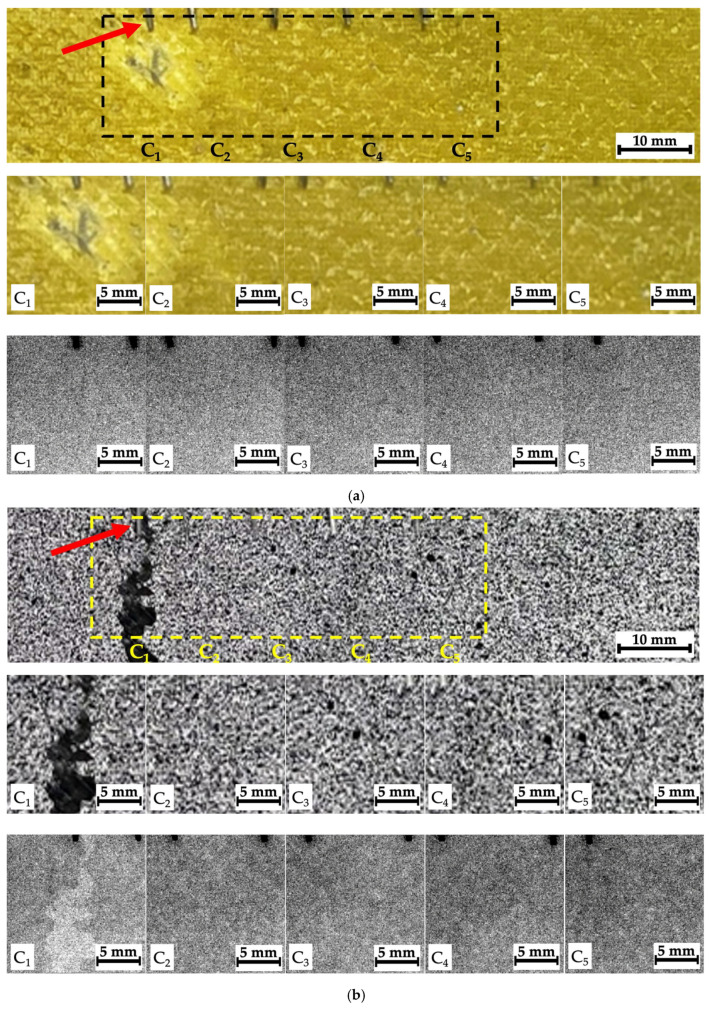

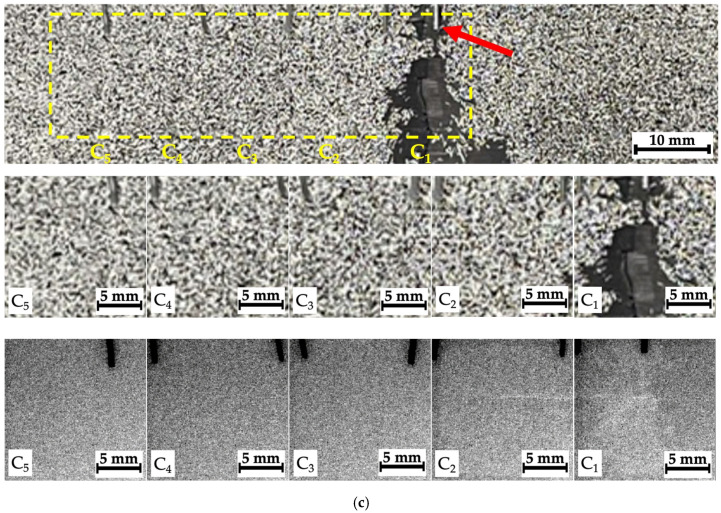
The regions of recording the radiographic images (montages) for composites #1 (**a**), #2 (**b**), and #3 (**c**); the arrows show office staples used as indicator marks.

Figure 7 presents images illustrating the locations of the investigated regions with indicator marks in the form of office staples (shown by arrows). Below, optical images of the composite surface within C1–C5 regions are shown with the corresponding radiographic data. It was quite difficult to detect any distinguishable “objects” reflecting damage caused by the impact of the striker (ball) or the drop weight in the unprocessed (raw) radiographic images. However, certain differences were evident for the studied samples. According to the authors, the reason was the specificity (difference in character) of the propagation of X-ray radiation in CFRPs (which have approximately similar radiographic density), but to the nature of the damage formed in them, associated with both the structure and different resistance to fracture under compression.

### 4.2. Composite #1, Separate Fragments

**Figure 8:**
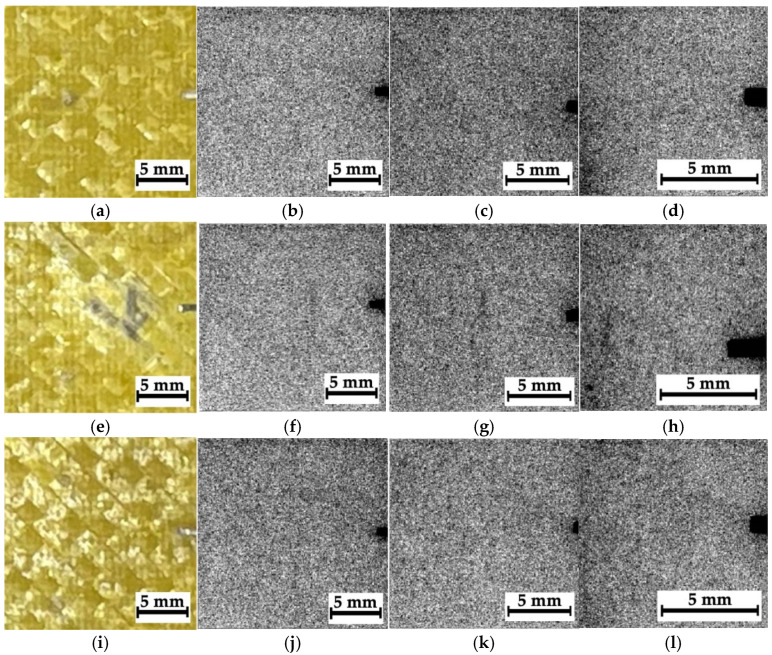
The optical images of regions N_1_–N_3_ (**a**,**e**,**i**) and the corresponding radiographic data for composite #1; the distances between the sample and the sensor are 39 mm (**b**,**f**,**j**), 160 mm (**c**,**g**,**k**), and 240 mm (**d**,**h**,**l**).

Figure 8 shows optical images and corresponding radiographic data for composite #1 (epoxy/AF), recorded at an accelerating voltage of 35 kV. The distance between the sample and the sensor was varied (39, 160, and 240 mm), changing the magnification (namely the sizes of the analyzed regions). Therefore, all three magnified radiographic images were used to detect discontinuities and in their qualitative and quantitative analysis.

According to Figure 8, the radiographic images did not reveal any noticeable visual difference between region N_2_ (Figure 8e–h), where the impact took place, and regions N_1_ and N_3_, located far from it (Figure 8a–d and Figure 8i–l, respectively). The reason could be the low spatial resolution of the sensor (about 50 μm) relative to the characteristic size of the damage. In addition, as shown in Figure 5b, after compression testing, the sample was characterized by pronounced delamination, so no differences in the radiographic data for regions N_1_–N_3_ could be observed. For effective radiographic inspection, defects should be oriented parallel to the X-ray beam, while in this case, the delamination was perpendicular to the axis of irradiation. Finally, one of the key challenges of the introscopy of low-contrast objects is the high level of noise/interference in the radiographic images. Since it was impossible to influence the first three factors within this study, the authors used algorithms described in Section 3 to solve the problem of improving radiographic data. Note that low residual compressive strength of 67.7 MPa was characteristic of composite #1.

**Figure 9:**
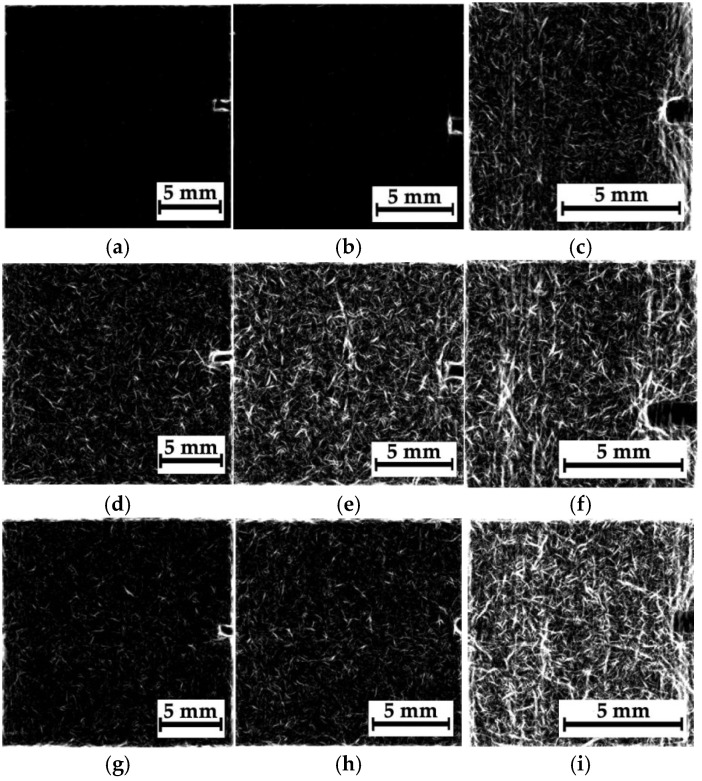
The phase variations of the radiographic images shown in Figure 8a–c; the distances between the sample and the sensor are 39 mm (**a**,**d**,**g**), 160 mm (**b**,**e**,**h**), and 240 mm (**c**,**f**,**i**).

**Figure 10:**
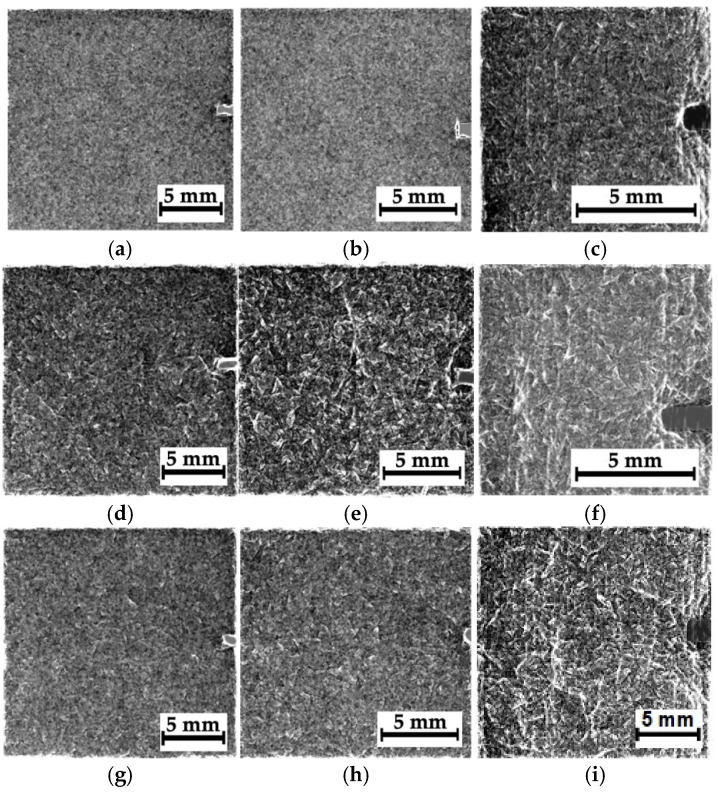
The phase variations of the processed radiographic images shown in Figure 8a–c; the distances between the sample and the sensor are 39 mm (**a**,**d**,**g**), 160 mm (**b**,**e**,**h**), and 240 mm (**c**,**f**,**i**).

Figure 9 presents the results of processing all the radiographic images, shown in Figure 8, in the phase-variation format. When calculating the phase variation using expression (7), the processing parameters used were *N* = 15, $\Psi =\frac{\pi }{15}$, and σ = 16. With increasing “magnification”, the degree of detail of the objects in the images was enhanced. According to the authors, this phenomenon was associated not only with the possible presence of damage but also with the structure of the layered CFRPs containing two different prepregs. Figure 10 shows the magnified images as “mixtures of the original images and their phase variations”. The use of both image processing methods increased their contrast via the detection of contours that reflected discontinuities formed during compression testing (Figure 5a).

In a preliminary discussion, the peculiarities of the analyzed radiographic data should be emphasized. In particular, the image quality is influenced by both discontinuities and the internal (including initial) structure of CFRPs. This is also due to the fact that the radiographic images are formed when X-rays passed through the entire thickness of the samples, while possible delamination may develop only locally. For this reason, the objects (identified in the images as contours) are not necessarily a result of damage development/presence. However, the detected “objects” (especially at higher magnifications) could result from fracture during compression testing since the sample structures were formed to be as homogeneous as possible during their fabrication. It is important to note that this assumption requires more detailed studies, including the implementation of alternative NDT methods, such as μCT.

The study was interested not only in visualizing the features of the internal structure but also in evaluating the efficiency of the proposed image processing methods, comparing the data for the investigated CFRPs. For this purpose, several metrics traditionally used in image-quality assessment tasks were employed. In particular, we calculated the values of the peak S-N ratio (PSNR) and the structure similarity index (SSIM) [85]. Table 4 provides a summary of the PSNR and SSIM values for the processed radiographic images (in fact, compared to the original ones, shown in Figure 8). The PSNR metrics were more informative since their values decreased with an increase in the number of visually observed contours (which can be interpreted as the number of objects in the images).

### 4.3. Composite #2, Separate Fragments

**Figure 11:**
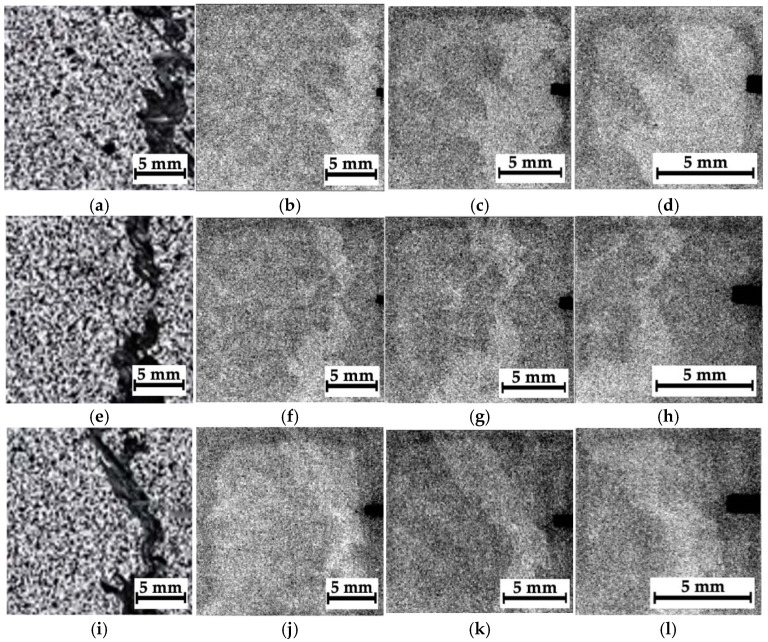
The optical images of regions N_1_–N_3_ (**a**,**e**,**i**) and the corresponding radiographic data for composite #2; the distances between the sample and the sensor are 39 mm (**b**,**f**,**j**), 160 mm (**c**,**g**,**k**), and 240 mm (**d**,**h**,**l**).

Figure 11a,e,i shows optical images and corresponding radiographic data for composite #2 (epoxy/CF). In this case, during the compression test, the main crack propagated across the entire sample, so its presence was characteristic of all regions, N_1_–N_3_ (Figure 11a,e,i). According to Figure 5c, the main crack propagation was accompanied by both delamination and microcracking. Thus, the “objects” visible in Figure 11 may also have been caused by this damage type (Figure 5c). Note that the residual compressive strength of composite #2 was 175 MPa.

**Figure 12:**
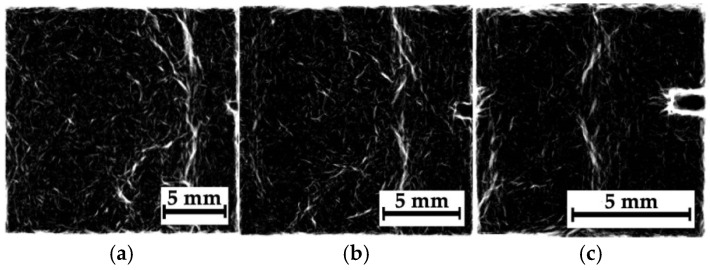

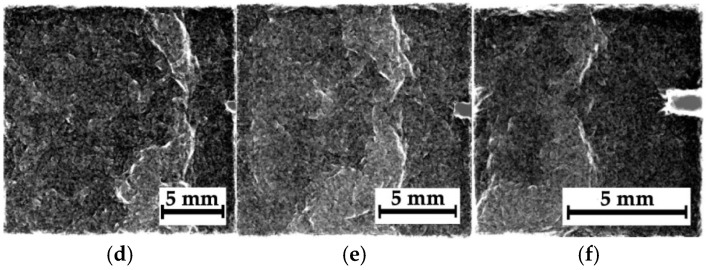
The results of processing of the radiographic images shown in Figure 11 as the phase variation ones (**a**–**c**), as well as the mixtures of the original images and the phase variations (**d**–**f**). The distances between the sample and the sensor are 39 mm (**a**,**d**), 160 mm (**b**,**e**), and 240 mm (**c**,**f**).

Since the damage types were similar for all three regions, N_1_–N_3_, the authors only reported the data for region N_2_, corresponding to the impact spot. Figure 12 shows the image processing results (Figure 11f–h) in the phase-variation format (Figure 11a–c) and as “mixtures of the original images and their phase variations” (Figure 11d–f). Unlike composite #1 (epoxy/AF), the degree of detail of the “objects” rather decreased with an increase in “magnification”. According to the authors, the reason was the change in dimensions of discontinuities, which were visualized quite clearly after the image processing procedure. This fact was also evidenced by the results of the quality metrics calculation for the images presented in Table 5. It can be seen that the metrics were enhanced somewhat as “magnification” increased. Most likely, this was caused by the fact that the number of “objects” (actually contours) decreased in the radiographic images, leading to reduced noise.

### 4.4. Composite #3, Separate Fragments

**Figure 13:**
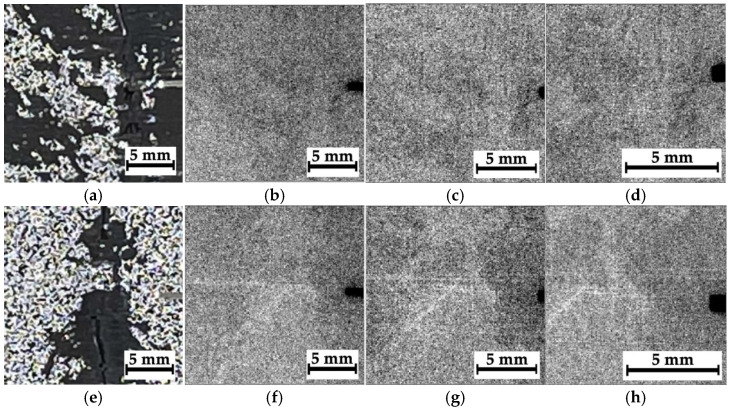

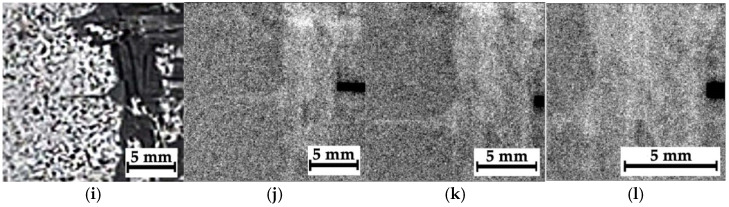
The optical images of regions N_1_–N_3_ (**a**,**e**,**i**) and the corresponding radiographic data for composite #3; the distances between the sample and the sensor are 39 mm (**b**,**f**,**j**), 160 mm (**c**,**g**,**k**), and 240 mm (**d**,**h**,**l**).

In composite #3 (PEEK/CF), the main crack during the compression test also propagated across the entire sample (Figure 5a). According to the visual assessment along the side face (Figure 5d), it was formed as a large number of localized microcracks. The acrylic paint layers peeled off from rather large areas (Figure 13), so the pattern of such discontinuities was similar to that in the digital image correlation method, indirectly reflecting pronounced damage. At the fractured area, the sample “swelled” to a significantly greater thickness compared to that for composite #2. As a result, the radiographic images were noticeably different for regions N_1_–N_3_ through the main crack path (Figure 13).

**Figure 14:**
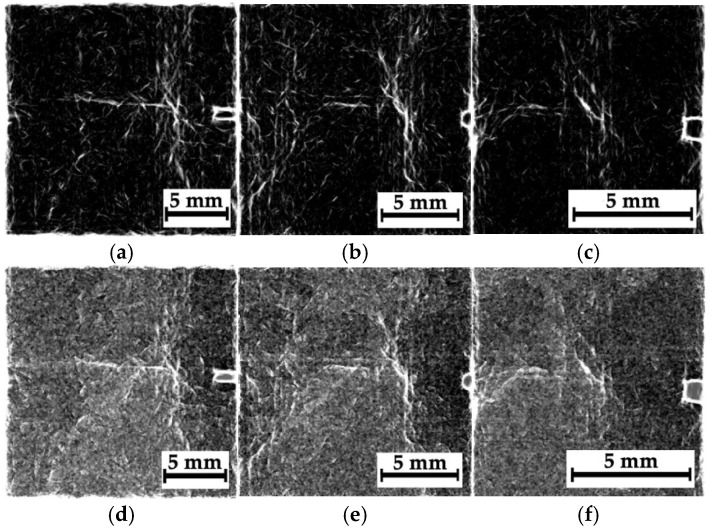
The results of processing the radiographic images, shown in Figure 13, as the phase variation images (**a–c**), as well as mixtures of both original images and phase variations (**d**–**f**); the distances between the sample and the sensor are 39 mm (**a**,**d**), 160 mm (**b**,**e**), and 240 mm (**c**,**f**).

The radiographic images were also processed only for region N_2_ through an analogy with the previous subsection; the results are shown in Figure 14. Compared with composite #2 (Figure 12), the images for all three “magnifications” appear similar, although the main crack is less clearly visible (probably due to the small sizes of its components compared to the spatial resolution of the sensor of 50 μm). Nevertheless, the objects (contours) observed in Figure 14 can be classified as discontinuities. Note that the residual compressive strength of composite #3 was equal to ~290 MPa.

Table 6 presents the calculated quality metrics of the processed radiographic images for composite #3 (PEEK/CF). For all three, both PSNR and SSIM parameters were characterized by close values of 15.4–19.7 and 0.78–0.84, respectively (they were 14.5–17.5 and 0.78–0.91, respectively, for composite #2). Thus, these ranges were comparable for the samples reinforced with carbon fibers and containing the main cracks formed under compression. At the same time, they differed for composite #1 (epoxy/AF) with the smaller discontinuities (its PSNR and SSIM values were 13.6–16.6 and 0.62–0.81, respectively). So, the phase variation pattern was mainly determined by the internal structure of composite #1 due to the smaller number of discontinuities and their sizes. For the more damaged composites #2 and #3, the “objects” (contours) in the processed radiographic images were specifically associated with their damage.

It should be noted that PSNR and SSIM metrics are typically used to compare optical images characterized by Gaussian noise in most cases. At the same time, the intensity and temporal distribution of quanta are described using the Poisson distributions, a special case of which is the Gaussian one. Respectively, for the analysis of the noise structure in the radiographic images, the results of their spectral analysis are presented in the Discussion section.

### 4.5. Composites #1–3, Montages of Fragments at Different Distances from the Impact Spots

**Figure 15:**
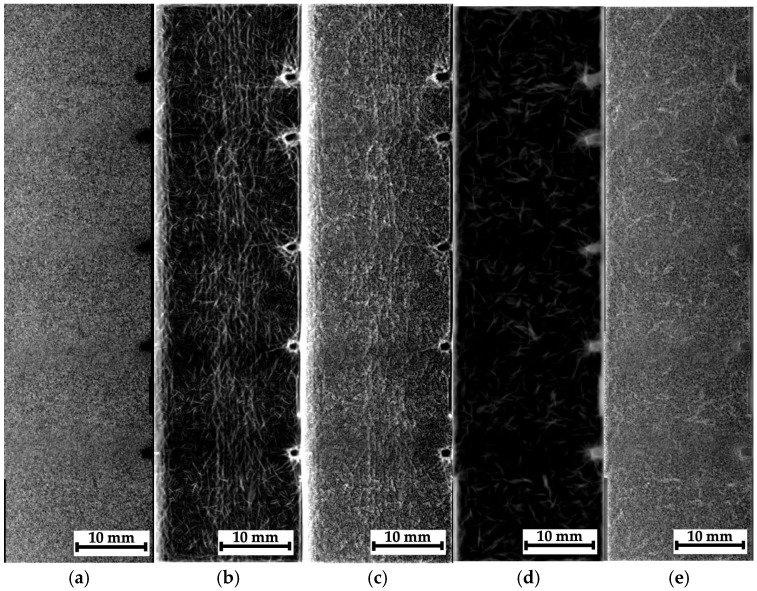
The processed radiographic images for composite #1: the preprocessed image (**a**), the phase variation (**b**), the sum of the phase variation and the pre-processed image (**c**), the phase congruency image (**d**), and the sum of the phase congruency and the pre-processed image (**e**).

**Figure 16:**
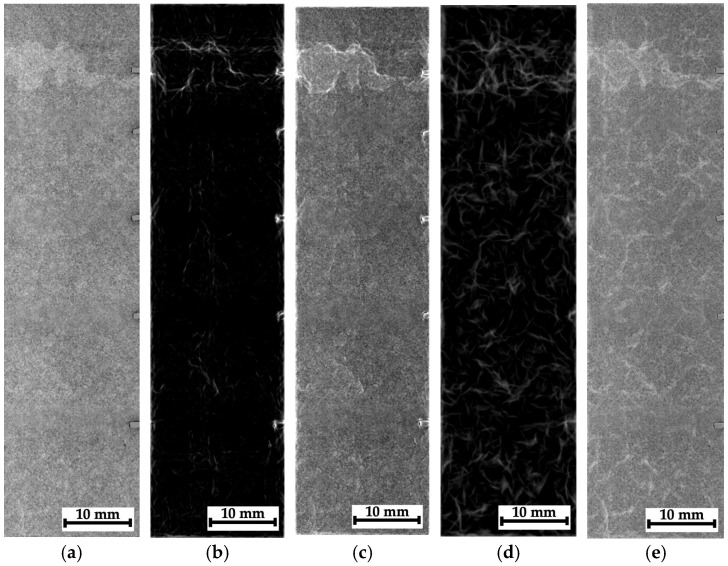
The processed radiographic images for composite #2: the pre-processed image (**a**), the phase variation (**b**), the sum of the phase variation and the pre-processed image (**c**), the phase congruency image (**d**), and the sum of the phase congruency and the pre-processed image (**e**).

**Figure 17:**
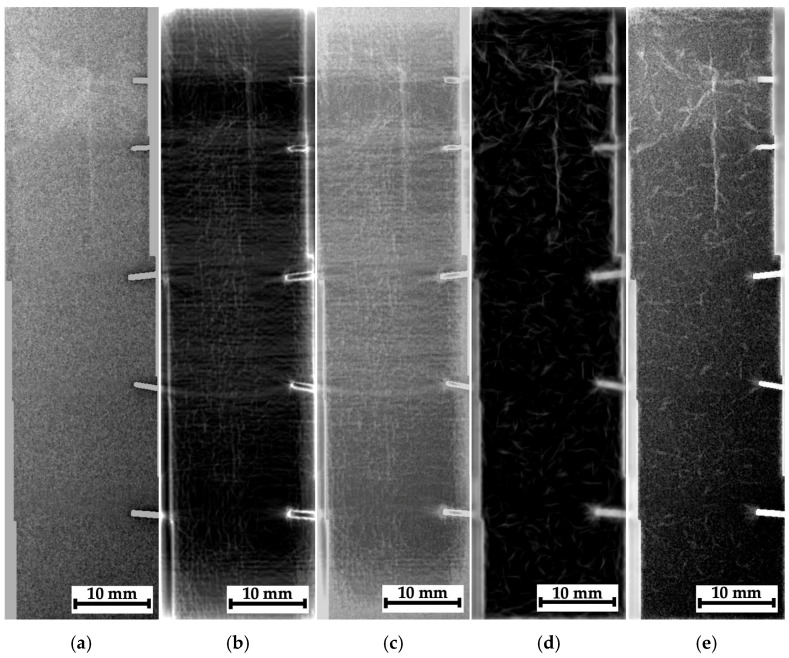
The processed radiographic images for composite #3: the pre-processed image (**a**), the phase variation (**b**), the sum of the phase variation and the pre-processed image (**c**), the phase congruency image (**d**), and the sum of the phase congruency and the pre-processed image (**e**).

As noted above, some montages of several stitched images were also recorded for composites #1–3. The recording scheme is shown in Figure 7a–c with the separate optical images and the corresponding radiographic data. In those cases, it was not possible to identify any “objects” due to the low contrasts. So, these radiographic data were processed according to the algorithms described in Section 3; the obtained results are shown in Figure 15, Figure 16 and Figure 17.

For composite #1 (epoxy/AF), where the damage is considered quasi-homogeneous interlayer delamination (Figure 5b), no discernible differences were detected in the pre-processed image at the impact spot and beyond (Figure 15a). For composites #2 (epoxy/CF) and #3 (PEEK/CF) with the main cracks formed during compression, both damage zones (crack propagation paths) were visible in similar images. However, its contrast was greater for composite #2 (Figure 16a) than that for composite #3 (Figure 17a).

A processing effect should be noted in the form of an increased concentration of contours along the left vertical edge of the images shown in Figure 15b,c. This should be interpreted as an artifact, rather than a consequence of the presence of discontinuities or the specificity of the internal composite structure. In addition, the phase congruency image (Figure 15d), as well as the sum of the phase congruency and the pre-processed image (Figure 15e), revealed the presence of needle-like “objects” (contours), indistinguishable in Figure 5b,c. The authors believe that these are not indicative of real discontinuities. This effect will be discussed in more detail in the corresponding section (Discussion).

The most obvious results of the image processing were obtained for composite #2 (Figure 16). First of all, it was possible to visualize the main crack. However, away from the main crack, no visible signs of discontinuities were detected in the phase-variation images (Figure 16b,c). This is generally consistent with the data presented in Figure 5c (the optical image of the side face of the sample), where the length of the maximum damaged area barely exceeded 10 mm. On the other hand, in the phase-congruency images (Figure 16d,e), similar to Figure 15d,e, a number of “objects” in the form of contours were detected, which, as the authors believe, reflected the specifics of the internal structure of composite #2 (epoxy/CF) but not discontinuities resulting from the growth of the main crack. Thus, the phase congruency method should be considered preferable for identifying large objects in radiographic images (with a low spatial frequency).

Figure 17 shows the processed radiographic images for composite #3 (PEEK/CF). As in the previous case, the main crack was successfully visualized. Figure 17b,c (the phase variations) presents some horizontal areas oriented parallel to it, which could be discontinuities caused by the impact loading of the sample (they were consistent with Figure 5d). In the phase congruency images (Figure 17d,e), which were more sensitive to “objects” from the low spatial frequency region, these areas were indistinguishable. However, Figure 17e most clearly visualizes both the main crack path and a vertically oriented object (which can be interpreted as a microcrack).

By analogy with the approach used above for quantitative assessment of the quality of the improved radiographic images, Table 7 shows the PSNR, SSIM, NIQE, and PIQE values for the phase variation montages (Figure 15b, Figure 16b and Figure 17b), the phase congruency (Figure 15d, Figure 16d and Figure 17d), and NIQE and PIQE values for the improved images (Figure 15a, Figure 16a and Figure 17a). For comparison, the same data are repeated for the single images at the impact spots, taken from Table 4, Table 5 and Table 6. For the montages, the PSNR values decreased relative to those for the single images of composites #1 (epoxy/AF) and #3 (PEEK/CF) due to the smaller numbers of “objects” (contours), contributing the most to the value of this parameter. Conversely, for the same reason, the PSNR value significantly increased for composite #2 (up to 26.9 relative to 14.4).

A less pronounced but generally similar trend was observed for the SSIM parameters, which remained virtually unchanged for composites #1 and #3 (0.81/0.82 and 0.75/0.78, respectively) but increased for composite #2 (0.94/0.78). NIQE and PIQE values exhibited the same trend, much like the relative metrics SSIM and NIQE. However, they are comparable with values for the non-processed images. Thus, the PSNR parameter should be considered more informative in terms of assessing the degree of improvement in the quality of radiographic images.

## 5. Discussion

One of the key challenges of this study was the impossibility (within the scope of the stated goal) of verifying the reliability of the radiographic data for low-contrast CFRPs with small-scale discontinuities and a sensor spatial resolution of ~50 μm (the exceptions were the general side views of the samples shown in Figure 5b–d). Depending on the applied algorithms, various “objects” (contours) in the processed radiographic images may be artifacts.

### 5.1. Spectrum Analysis

The approach used by the authors for a quantitative assessment of the processed images was borrowed from conventional methods of comparing the quality of optical images, where the main noise source (interference) is the noise from the CCD (charge-couple device) or CMOS (complementary metal oxide semiconductor) sensors, typically characterized by Gaussian distributions. In this study, the processed images were formed by accumulating (counting) quanta, while the condition for completing the recording process was reaching their number of 10,000. From the standpoint of understanding both the image contents and the effects of their processing, identifying the nature of this noise is important, which can be achieved via a 2D spectral analysis.

**Figure 18:**
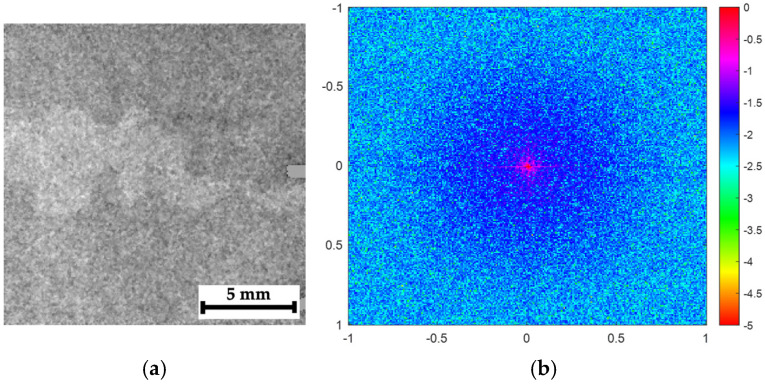

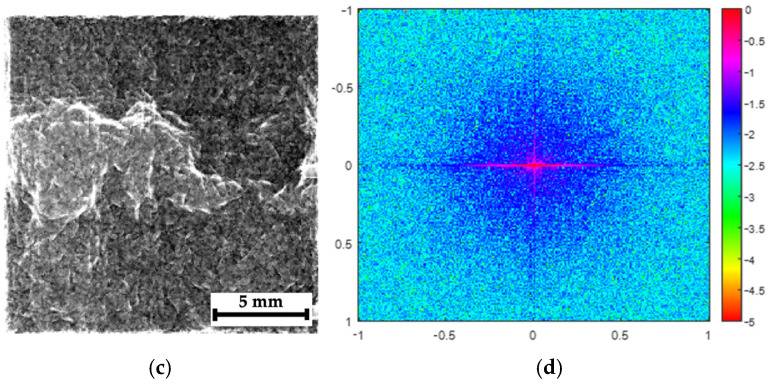
The results of the spectral analysis of the radiographic images for composite #2 (fragment C1), (**a**) the pre-processed image, (**b**) its 2D Fourier spectrum, (**c**) a mixture of the original image and its phase variation, and (**d**) its 2D Fourier spectrum; the color palette of the spectra is presented in decibels, while the frequency scale is in relative units (normalized to half the Nyquist frequency).

Figure 18a shows a radiographic image of fragment C1 (Figure 7c) and its 2D Fourier spectrum obtained using the “Mathlab” software package version R2023b. The spectrum energy was noticeably blurred across the entire range of spatial frequencies (Figure 18b). For the processed image (a mixture of the original image and its phase variation shown in Figure 18c), the presence of a clearly distinguishable area of discontinuities manifests in the 2D Fourier spectrum as a concentration of energy in the low-frequency range (Figure 18d).

**Figure 19:**
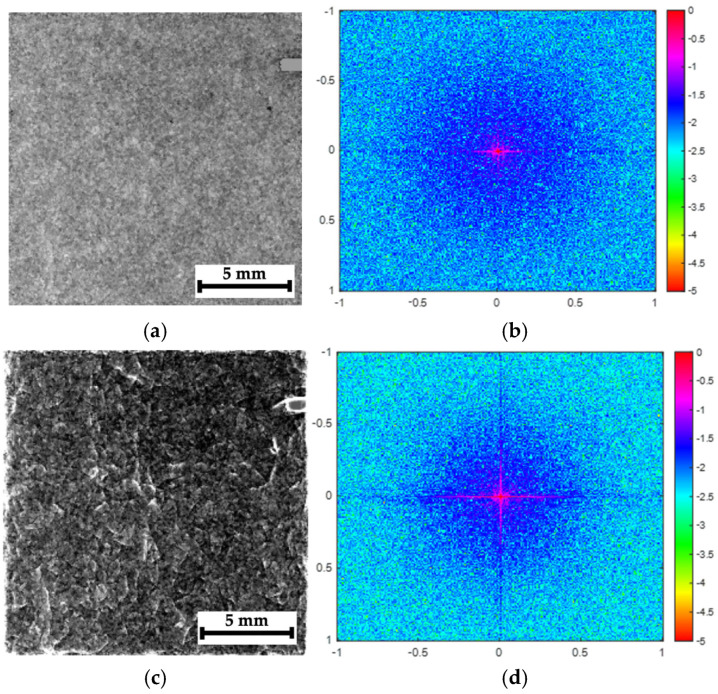
The results of the spectral analysis of the radiographic images for composite #2 (fragment C5), (**a**) the pre-processed image, (**b**) its 2D Fourier spectrum, (**c**) a mixture of the original image and its phase variation, and (**d**) its 2D Fourier spectrum; the color palette of the spectra is presented in decibels, while the frequency scale is in relative units (normalized to half the Nyquist frequency).

If the analyzed image does not contain a clearly visible damaged area, such as in Figure 19a (fragment C5 in Figure 7c), the 2D Fourier spectrum on the raw radiographic image (Figure 19b), characterized by a low S-N ratio, is similar to that shown in Figure 18b. The 2D Fourier spectrum of the processed image (a mixture of the original image and its phase variation shown in Figure 19c) reflects details of the internal composite structure (but not discontinuities in it since, according to the authors, they are absent or minimal). The 2D Fourier spectrum also indicates a concentration of energy in the low-frequency range (Figure 19c).

Thus, regardless of the nature of detected objects, the image processing, performed by adding the phase-variation pattern after their pre-processing, enables the elimination of a noticeable portion of the high-frequency noise, making the images clearer and more contrasting.

### 5.2. The Noise Immunity Study

**Figure 20:**
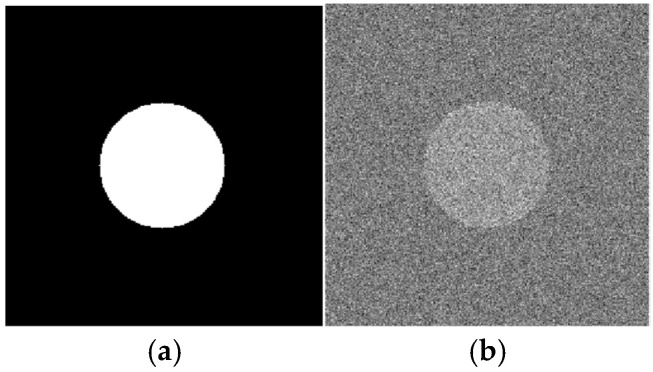
The model (**a**) and processed (**b**) images of the circle; noise at *ρ* = 1.

To better understand the results of processing the radiographic images described above, the noise immunity of the phase congruency and the phase-variation algorithms were investigated on a model image of a circle contour. Images of the circle (Figure 20a) and an image of Gaussian white noise were synthesized using the “Mathlab” software package version R2023b. A weighted sum of both the circle and noise images with different *ρ* signal-to-noise ratios (Figure 20b) was the input parameter of the processing algorithms.

**Figure 21:**
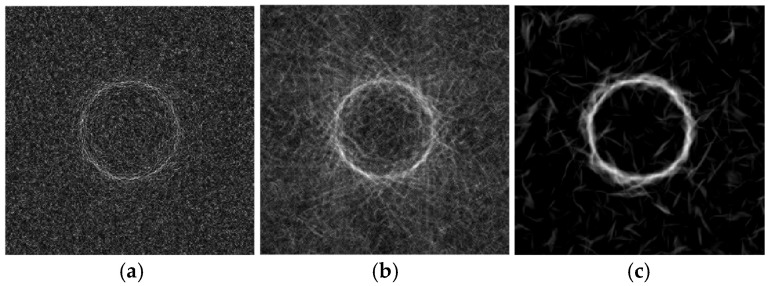
The results of the phase variation (**a**), improved phase variation (**b**), and congruency (**c**) functions applied for the circle image with noise at *ρ* = 1.

For all images with noise, the phase congruency (M = 63, N = 6, $\sigma_{\omega }$ = 16, $\sigma_{\theta }$ =1.7), the phase variation, and the improved phase variation (N = 15, $\Psi =\frac{\pi }{15}$, σ = 16) were calculated. An example of the results is shown in Figure 21.

**Figure 22:**
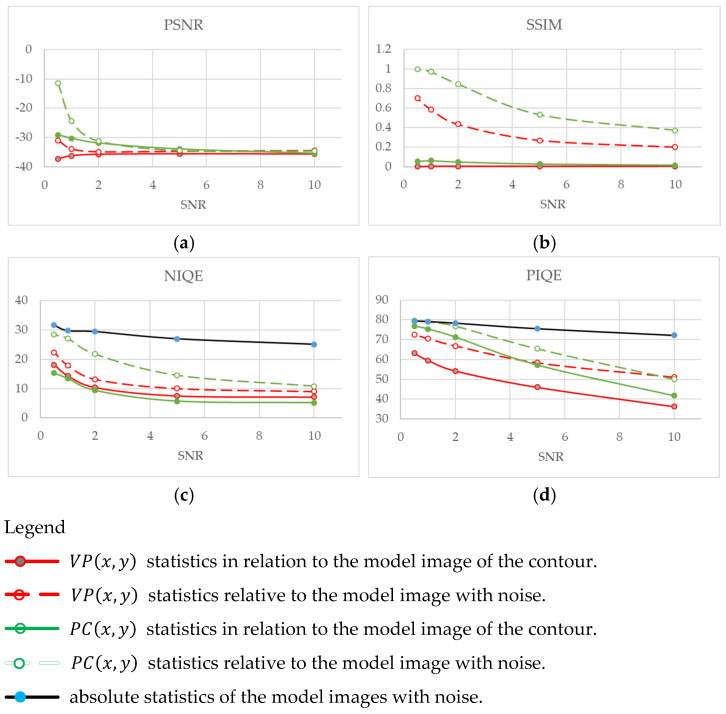
The results of the study of the algorithms for detecting contours for noise immunity by calculating various metrics: PSNR (**a**), SSIM (**b**), NIQE (**c**) and PIQE (**d**).

Consistent with the conclusions noted above, it is evident that the phase-variation function highlights high-frequency elements (Figure 21b), while phase congruency reflects low-frequency objects (contours, Figure 21c). It should be noted that the bright needle-like areas in Figure 22c are, to some extent, similar to those in Figure 15e, Figure 16e and Figure 17e. Consequently, this may indicate that their origin is the high noise level of the original radiographic images. This phenomenon, however, does not diminish the effectiveness of the applied approach to image processing in highlighting low-spatial-frequency objects.

The obtained model images were used to calculate the PSNR and SSIM relative statistics (full-reference quality metrics), as well as the absolute statistics (no-reference quality metrics), in particular, the natural image quality evaluator (NIQE) and perception-based image quality evaluator (PIQE) [88,89]. Initially, the metrics were assessed according to the ratio of the phase variation and congruency functions to the model image of the contour. Then, the basis was the ratio of the sum of the processed images and their *PC* and *VP* functions.

As expected, almost all PSNR values tended toward a constant level of 34 dB (Figure 22a). An exception was the contour image with the applied variation function, the PSNR value of which decreased with an increasing the S-N ratio (enhanced in the dB scale). This result requires a separate study, as it most likely indicates the ambiguity of the PSNR metrics applied to the investigated images.

Typically, the SSIM values, as statistics of the similarity of two images, are interpreted as “similar” when close to one and “different” when close to zero. In the studied cases, the SSIM values must be interpreted differently: closeness to one means the images are unchanged, while tending to zero means a significant change. Therefore, the processed images are characterized by higher SSIM values with increasing noise levels, i.e., they possess negligible changes (Figure 22b). The comparison of the edge and phase function images turns out to be practically indistinguishable for the SSIM metrics and close to zero for the entire range of the S-N ratios.

The absolute metrics NIQE and PIQE both assess image quality without a comparison with the standard, and they are more preferable for research on real radiographic data. Their analysis showed an adequate and explainable result (Figure 22c–d): with an increase in the S-N ratio, all these absolute metrics decreased, reflecting an improvement in image quality. However, if the analysis of the sum of the images processed using the phase functions showed better quality after the phase-variation function was applied for both metrics, then both metrics, NIQE and PIQE, revealed reverse preferences when contour images (functions) were analyzed. The advantages of the PIQE metrics include the proximity of the quality assessment values of both contour and processed images, which makes it the most preferable among those applied.

It should be noted that the radiographic registration mode implemented in this study provides an acceptable level of spatial resolution and penetration ability. Consequently, it can be used in systems for inspecting products of significant length. The obtained results enable an expectation of a successful solution to this class of problems, while automated digital X-ray radiography is expected to remain among the most efficient and reliable NDT means for the inspection of components made from CFRP.

Before coming to conclusions, the following limitations, as well as prospects, of the study are to be postulated.

The residual strength is a “standard” measure of the bearing capacity of a composite plate after an impact test. It is a function of multiple parameters, including constituents of a composite, adsorbed impact energy, a type and parameters of impact tests, damaging and cracking pattern, etc. Thus, there should be a certain correlation between residual strength and the dimension of the main crack, but it is not a linear or direct correspondence. This issue was not discussed in the paper; however, the authors will focus on this topic in the forthcoming research.

When processing the model images simulating the radiographic images, the applied algorithms highlighted non-existent objects at the low S-N ratios. For this reason, it is necessary to develop new approaches to data interpretation that are capable of assessing the realism of the identified discontinuities.

The utilized approach to radiographic image improvement is not unique. The set of image-processing algorithms used can be extended, depending on the pattern of noising or distortion. But the use of phase congruency and phase variation instead of popular algorithms for edge detection is a real novelty. It was illustrated in the successful example of detecting low-contrast objects when the signal-to-noise ratio was below unit.

The authors associate further research prospects with the use of GaAs sensors, testing the installation for detecting other types of discontinuities and structural inhomogeneities in CFRPs (laminates), and selecting rational radiographic recording modes that provide images with higher S-N ratios. Of great practical relevance is testing the developed hardware/software tool with real components and real damage. Thus, the sensitivity of the developed method will be assessed with a variation in materials, geometry, irradiation doses, the type and dimension of damage, etc.

## 6. Conclusions

An improvement in the radiographic images of the CFRPs after subsequent impact and compression loading was achieved using the developed algorithms. The following conclusions were drawn.

1. For composite #1 (epoxy/AF) subjected to the “high-velocity” steel-ball impact with subsequent compression loading, it was not possible to detect discontinuities and/or the main crack. The reason was the orientation of the extended zone of interlayer delamination being perpendicular to the irradiation axis, while discontinuities caused by the impact could not be visualized due to their negligible dimensions. After the drop-weight impacts and subsequent compression loading of composites #2 and #3 (epoxy/CF and PEEK/CF, respectively), the main cracks were formed in their central parts. For composite #2, the damage was less localized and represented in a set of both longitudinal delamination and transverse microcracks. This area was reliably detected in the processed radiographic images; moreover, it achieved the highest contrast compared to that for composite #3, which was similar but lower. These slight dimensional discontinuities were difficult to reveal in the processed radiographic images, which were characterized by a lower contrast as compared to composite #2.
2. The analysis of the combined radiographic images (montages) has shown that the “objects” (contours) detected at a distance from the main crack are mainly associated with the specifics of the internal composite structure, rather than with damage formed during compression testing. The reasons are the interaction of X-ray radiation with the material and artifacts caused by a low S-N ratio. Another challenge is the orientation of discontinuities perpendicular to the X-ray beam axis, in addition to their small size and low contrast in the CFRP samples. The potential way to solve the problem might be a variation in the X-ray irradiation angle.
3. Phase-variation and phase-congruency methods were employed to highlight low-contrast objects in radiographic images. In real images of the aforementioned composites, the phase-variation procedure showed its efficiency in detecting small objects (with a high spatial frequency), while the phase-congruency method is preferable for highlighting large objects (with a low spatial frequency).
4. To assess the efficiency of the implemented image processing methods, full- (PSNR and SSIM) and no-reference (NIQE and PIQE) quality metrics were used. In the analysis of the model images, the SSIM metric exhibited low sensitivity to changes, while the PSNR parameter was the most indicative (with an S-N ratio greater than one unit), confirming an increase in the image contrast and a decrease in the noise level. In contrast to the PSNR metric, the NIQE and PIQE parameters enable the correct assessment of image quality even with an S-N ratio of less than a unit. For the processed radiographic images, the low noise levels were clearly demonstrated in the 2D Fourier spectra, which showed a shift in the main energy component towards the low-spatial frequency domain.

## Figures and Tables

**Table 1:** The results of the impact test and the residual compressive strength value for composite #1.

Thickness, mm	Striker Velocity Before Impact, m/s	Striker Kinetic Energy Before Impact, J	Impact Energy Per Millimeter of Thickness, J/mm	Elasticity Modulus (Stiffness), GPa	Compression Strength After Impact, MPa
3.2	90.8	49.1	15.3	30.1	67.7

**Table 2:** The test results for composite #2 (velocity = 3.5 m/s).

Thickness, mm	Impact Energy, J	Impact Energy Per Millimeter of Thickness, J/mm	Absorbed Energy, J	Peak Force, N	Damage Initiation Force, N	Damage Initiation Energy, J	Compression Strength After Impact, MPa
4.72	63.7	13.5	43.3	15,630.2	4803.1	3.0	175.7

**Table 3:** The test results for composite #3 (Velocity = 3.34 m/s).

Thickness, mm	Impact Energy, J	Impact Energy Per Millimeter of Thickness, J/mm	Absorbed Energy, J	Peak Force, N	Damage Initiation Force, N	Damage Initiation Energy, J	Compression Strength After Impact, MPa
4.42	29.9	6.8	20.3	10,020.9	7481.1	10.7	290.8

**Table 4:** The MSE/PSNR/SSIM values for the processed radiographic images for composite #1.

Statistics	Region	L, mm		
		39	160	240
PSRN	N_1_	32.7377	32.6135	16.2886
	N_2_	16.6318	13.6818	14.0694
	N_3_	20.6828	21.6504	10.6309
SSIM	N_1_	0.9871	0.9922	0.7665
	N_2_	0.8175	0.6802	0.6244
	N_3_	0.8993	0.8678	0.5423
NIQE	N_1_	12.0365	12.0697	12.0860
	N_2_	12.9296	12.0985	12.4374
	N_3_	12.7888	12.2750	12.2109
PIQE	N_1_	50.2905	50.2666	54.4745
	N_2_	54.9133	56.4914	56.0223
	N_3_	51.9405	57.1815	56.0704

**Table 5:** The MSE/PSNR/SSIM values for the processed images for composite #2, region N_2_.

Statistics	L, mm		
	39	160	240
PSNR	14.4486	16.6805	17.5288
SSIM	0.7846	0.8291	0.9118
NIQE	9.8329	10.8618	11.3997
PIQE	47.9991	47.9058	46.1047

**Table 6:** The MSE/PSNR/SSIM values for the processed images for composite #3, region N_2_.

Statistics	L, mm		
	39	160	240
PSNR	15.4689	19.7381	17.1512
SSIM	0.7828	0.8049	0.8476
NIQE	11.4462	11.8984	12.2921
PIQE	52.4639	48.5027	45.9538

**Table 7:** The PSNR/SSIM/NIQE/PIQE values for the improved images, composites #1–3.

Method	PSNR/PSNR	SSIM/SSIM	NIQE	PIQE
ROW (composite #1)			11.406	58.340
Phase variation	13.998/16.631 (Table 4)	0.812/0.818 (Table 4)	9.847	50.699
Phase congruence	23.364	0.962	10.805	57.096
ROW (composite #2)			10.980	55.381
Phase variation	26.933/14.449 (Table 5)	0.946/0.785 (Table 5)	8.137	37.192
Phase congruence	33.077	0.983	9.560	46.025
ROW (composite #3)			10.192	53.619
Phase variation	11.798/15.468 (Table 6)	0.754/0.782 (Table 6)	8.595	40.810
Phase congruence	20.993	0.945	9.210	51.369

## Data Availability

The data presented in this study are available upon request from the corresponding author.
